# Bullöses Pemphigoid im Säuglingsalter

**DOI:** 10.1111/ddg.15745_g

**Published:** 2025-09-15

**Authors:** Jovine Ehrenreich, Linda Golle, Thomas Lange, Enno Schmidt, Burkhard Kreft

**Affiliations:** ^1^ Universitätsklinik und Poliklinik für Dermatologie und Venerologie Universitätsklinikum Halle (Saale) Martin‐Luther‐Universität Halle‐Wittenberg, Halle (Saale); ^2^ Universitätsklinik und Poliklinik für Pädiatrie Universitätsklinikum Halle (Saale) Martin‐Luther‐Universität Halle‐Wittenberg, Halle (Saale); ^3^ Klinik für Dermatologie Allergologie und Venerologie Universitätsklinikum Schleswig‐Holstein Campus Lübeck Lübecker Institut für Experimentelle Dermatologie (LIED) Universität zu Lübeck, Lübeck

Sehr geehrte Herausgeber,

Ein 5 Monate alter Säugling afghanischer Herkunft wurde mit einer zwei Monate andauernden Vorgeschichte zunehmend urtikarieller Plaques, prall gespannter Blasen und ausgedehnter Erosionen an Rumpf und Extremitäten bei starkem Juckreiz vorgestellt. Initial waren die periumbilikale Region, der untere Rücken sowie Palmae und Plantae betroffen (Abbildung 1a–c). Die Schleimhäute zeigten keinen Befall.

**ABBILDUNG 1:**
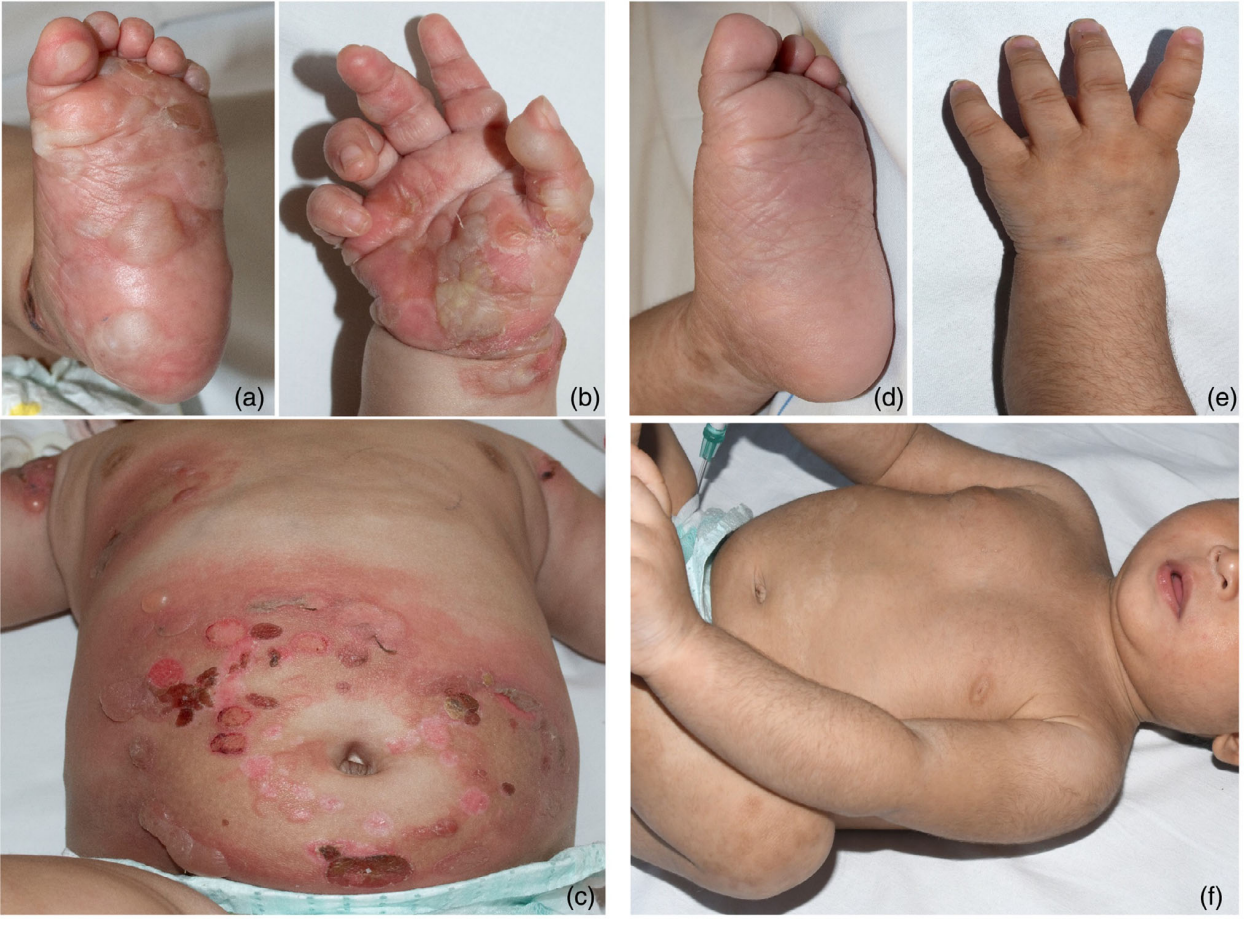
Klinisches Erscheinungsbild bei Therapiebeginn und nach der Behandlung. (a–c). Ausgeprägte Blasenbildung auf erythematöser Haut mit Schwerpunkt auf den Plantae, den Palmae und dem Rumpf. (d–f) Vollständige Remission nach 15 Zyklen IVIG und nach Beendigung der Therapie mit Dapson, systemischem und topischem GCS.

Weder in der Familienanamnese noch in der Schwangerschaftsanamnese gab es Auffälligkeiten. Eine Sechsfachimpfung (Diphtherie, Keuchhusten, Tetanus, Poliomyelitis, Hepatitis B, *Haemophilus influenzae* Typ B) sowie eine Pneumokokken‐ und Rotavirenimpfung wurden 4 Wochen vor Auftreten der Effloreszenzen durchgeführt.

Eine Biopsie einer frischen Blase ergab histologisch eine subepidermale, spongiforme, eosinophilenreiche Dermatitis. In der direkten Immunfluoreszenz eines periläsional entnommenen Hautbioptats konnte eine lineare Fluoreszenz bei Beschichtung mit antihumanem IgG (n‐Muster) und Komplement C3 an der Basalmembran nachgewiesen werden (Abbildung 2).

**ABBILDUNG 2:**
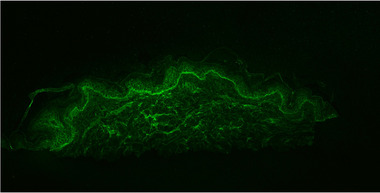
Die direkte Immunfluoreszenz zeigt eine lineare Fluoreszenz bei Beschichtung mit antihumanem IgG (n‐Muster) und Komplement C3 an der Basalmembran.

Eine indirekte Immunfluoreszenz auf mit NaCl‐separierter menschlicher Spalthaut zeigte zirkulierende IgG‐ und in geringerem Maße IgA‐Autoantikörper mit Bindung am Dach der künstlichen Blase. Ein ELISA mit rekombinanter BP180 NC16A‐Domäne war mit 1360 U/ml hoch positiv (Normwert < 20 U/ml).

Zusammenfassend diagnostizierten wir ein bullöses Pemphigoid (BP) im Säuglingsalter.

Zum Zeitpunkt der Erstvorstellung war der Säugling bereits seit 4 Wochen mit Dexamethason 0,4 mg/kg Körpergewicht (KG) per os und topischen Glukokortikosteroiden (GCS) Klasse II auf der betroffenen Haut behandelt worden. Nach der Vorstellung des Falls in der Fallkonferenz zu bullösen Autoimmundermatosen der Klinik für Dermatologie, Universitätsklinikum Schleswig‐Holstein, Campus Lübeck, im Zentrum für seltene Erkrankungen, wurde die systemische GCS‐Therapie auf Hydrocortison 0,5 mg/kg KG per os umgestellt. Zusätzlich initiiert wurden Dapson 2 mg/kg KG per os, Cetirizinsaft 0,6 mg/kg KG und intravenöse humane Immunglobulingabe (IVIG) in einer Dosierung von 2 g/kg KG alle 4 Wochen über je 3 Tage verabreicht. Die lokale Therapie wurde auf ein topisches GCS der Klasse III in Kombination mit Octenidinhydrochlorid auf dem gesamten Integument eskaliert. Unter der Therapie mit Dapson war der Methämoglobinspiegel mit 9,6 % erhöht (normal < 1,5 %), jedoch asymptomatisch und daher ohne klinische Relevanz. Ein GCS‐induziertes iatrogenes Cushing‐Syndrom ließ sich nicht vermeiden. Dieses zeigte sich serologisch durch stark erniedrigte Cortisol‐ und ACTH‐Spiegel am Morgen und klinisch durch eine typische Fettumverteilung.

Unter den therapeutischen Maßnahmen bot sich zunächst eine deutliche Befundprogression in zuvor nicht betroffenen Hautarealen mit Schwerpunkt auf den Extremitäten, dem Gesicht und den Genitalien. Bei unveränderter Fortführung der topischen und systemischen Therapie konnte schließlich nach dem dritten Zyklus der IVIG‐Therapie ein deutlicher Rückgang der Krankheitsaktivität mit Sistieren der Blasenbildung und des Pruritus objektiviert werden. Der klinische Verlauf wurde anhand des *BP Disease Area Index* (BPDAI) und der *BPDAI Pruritus‐Komponente* gemessen.^1^

Das topische GCS und Dapson konnten im Verlauf und bei klinischer Krankheitskontrolle ausgeschlichen und vollständig abgesetzt werden. Aufgrund der Entwicklung des oben erwähnten GCS‐induzierten iatrogenen Cushing‐Syndroms und um eine Addison Krise zu vermeiden, musste das systemische GCS langsam reduziert werden und wurde folglich erst einige Monate nach Absetzen des Dapsons vollständig beendet. Nach dem 10. Zyklus der IVIG‐Therapie konnten immunserologisch nur noch geringe Anti‐BP180‐Antikörperspiegel nachgewiesen werden (36 U/ml). Da auch nach sukzessiver Verlängerung der IVIG‐Intervalle keine erneuten klinischen Manifestationen auftraten, wurde die Therapie nach dem 15. IVIG‐Therapiezyklus beendet (Abbildung 3). Die komplette Remission des BP besteht heute, 24 Monate nach Therapiebeginn, auch nach Beendigung aller Therapiemaßnahmen fort (Abbildung 1d–f).

**ABBILDUNG 3:**
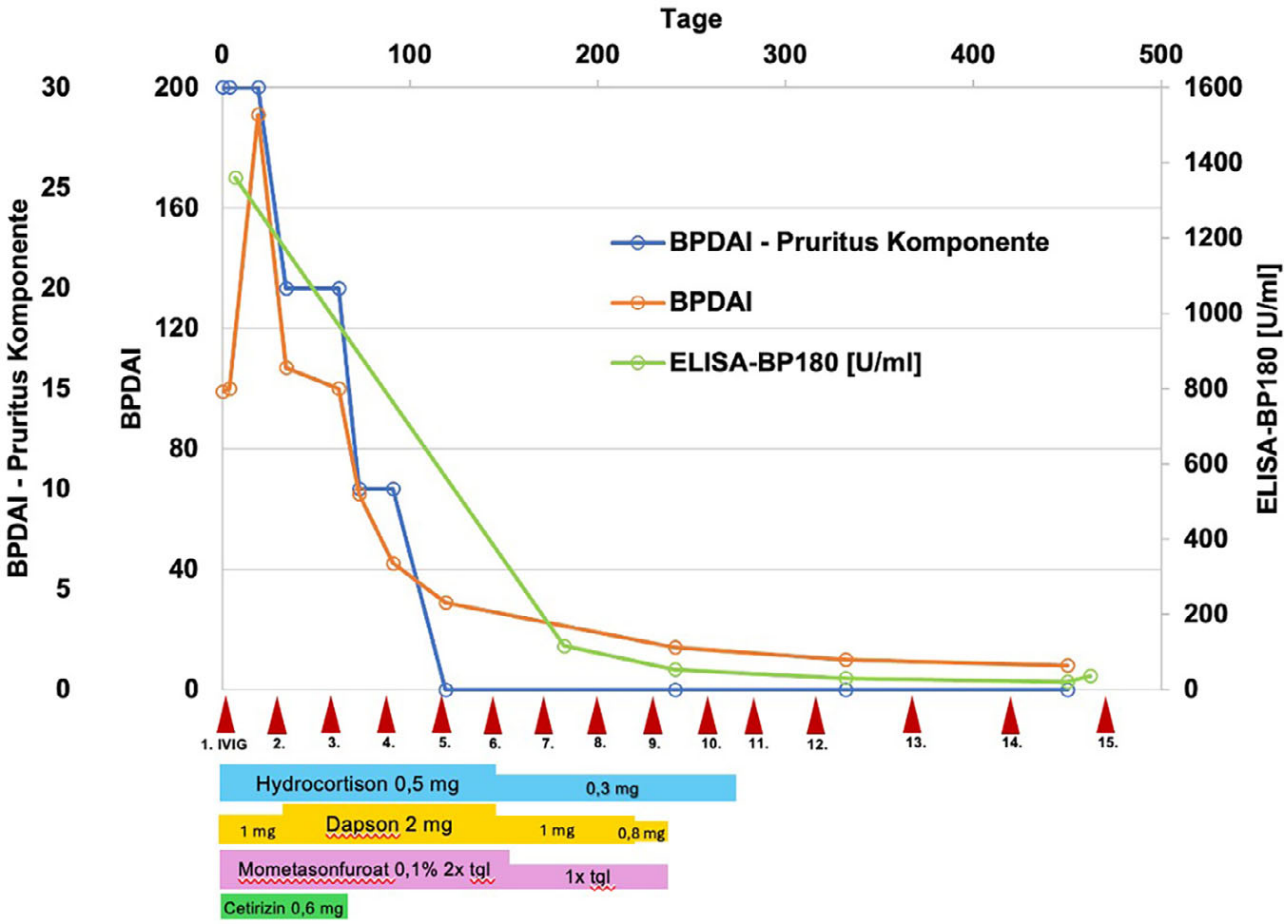
Grafik zur Veranschaulichung des klinischen Verlaufs, erhoben mittels dem *BP Disease Area Index* (BPDAI) und der *BPDAI Pruritus Komponente*, sowie Verlauf der ELISA‐BP180‐Werte und der Therapieanpassungen. Die gemessene Zeitspanne beginnt mit der in unserer Klinik initiierten Therapie. Dosierung angegeben in mg/kg KG sowie Häufigkeit der Anwendung pro Tag für die topische Therapie.

Das BP ist eine erworbene blasenbildende Autoimmundermatose mit subepidermaler Spaltbildung, die durch das Vorhandensein von IgG‐Autoantikörpern gegen die beiden hemidesmosomalen Strukturproteine der Basalmembranzone BP180 (Typ‐XVII‐Kollagen) und BP 230 gekennzeichnet ist. Die Erkrankung betrifft in der Regel ältere Menschen, mit einer Inzidenz von 190 Fällen pro 1 Million Einwohner bei Personen über 80 Jahren.^2^ Im Kindesalter ist ein bullöses Pemphigoid äußerst selten und weist in Deutschland eine Prävalenz von etwa 5 Fällen pro 1 Million Menschen unter 18 Jahren auf.^3^ Bisher wurden in der Literatur weniger als 100 Fälle beschrieben.^4^

Im Kindesalter wird ein Erkrankungsgipfel um den 4. Lebensmonat sowie um das 8. Lebensjahr beobachtet.^5^ Klinisch stehen im Säuglingsalter Hautsymptome an Palmae, Plantae und im Gesicht im Vordergrund. Im Säuglingsalter sind zunehmend auch die Mund‐ und Genitalschleimhaut betroffen.^3^, ^6^, ^7^

Die auslösenden Faktoren für das BP im Kindesalter sind noch nicht abschließend geklärt. Es gibt Fallberichte, die einen Zusammenhang mit Impfungen oder einer vorangegangenen Infektion vermuten lassen.^8^, ^9^, ^10^ Da im Kindesalter jedoch regelmäßig geimpft wird, ist ein eindeutiger epidemiologischer Zusammenhang derzeit nicht nachweisbar.^11^ Pathogenetisch ist jedoch die immunologische Demaskierung eines subklinischen BP durch Impfungen oder Infektionen denkbar.^11^ Im Vergleich zum BP bei Erwachsenen konnte bisher kein Zusammenhang mit Medikamenten oder malignen Grunderkrankungen festgestellt werden.^3^, ^7^

Die Differenzialdiagnosen sollten eine lineare IgA‐Dermatose, Dermatitis herpetiformis und Epidermolysis bullosa acquisita einschließen, die im Kindesalter gehäuft vorkommen.^11^ Weitere Differenzialdiagnosen sind Epidermolysis bullosa hereditaria, bullöse Mastozytose, bullöse Impetigo, Krätze, Insektenstichreaktionen, Porphyrien, atopische Dermatitis und medikamenteninduzierte Dermatosen.^6^, ^7^, ^11^

Trotz der anfänglich beeindruckenden klinischen Manifestationen hat das BP bei Kindern eine gute Prognose mit meist vollständiger Remission der Krankheitsaktivität innerhalb von Wochen bis Monaten und einer durchschnittlichen Krankheitsdauer von 14 Monaten.^12^

Ein Behandlungsalgorithmus wurde von Schwieger‐Briel et al.^7^ vorgeschlagen, der auf Behandlungsempfehlungen für Erwachsene basiert: konsequente topische Behandlung mit GCS der Klasse II bis III sowohl auf betroffenen als auch nicht betroffenen Hautarealen. Wenn eine systemische Therapie angezeigt ist, sollte zunächst systemisches GCS in einer Dosierung von 0,5 mg/kg KG verabreicht werden. Bei fehlendem Ansprechen kann die Therapie mit Dapson, Mycophenolatmofetil, IVIG oder Azathioprin erweitert werden, für Letzteres liegen jedoch bislang unzureichende Daten vor.^7^, ^11^, ^13^ Bei refraktärem Fortschreiten der Erkrankung trotz Eskalation der Therapie über 6–8 Wochen wird eine Behandlung mit Rituximab empfohlen.^4^, ^7^

Mit unserem Fallbericht möchten wir darauf hinweisen, dass das BP auch im Säuglings‐ oder Kindesalter auftreten kann. Andere blasenbildende Hauterkrankungen des Kindesalters müssen bei der Differenzialdiagnose abgegrenzt werden. In den meisten Fällen kommt es innerhalb einiger Monate zu einer vollständigen Remission der Erkrankung. Die Behandlung ist anspruchsvoll und herausfordernd und sollte interdisziplinär unter Beachtung potenzieller Nebenwirkungen abgestimmt werden.

## DANKSAGUNG

Open access Veröffentlichung ermöglicht und organisiert durch Projekt DEAL.

## INTERESSENKONFLIKT

Keiner.
